# Promising Genetic Biomarkers of Preclinical Alzheimer's Disease: The Influence of *APOE* and *TOMM40* on Brain Integrity

**DOI:** 10.1155/2012/421452

**Published:** 2012-04-09

**Authors:** Beata Ferencz, Sari Karlsson, Grégoria Kalpouzos

**Affiliations:** Aging Research Center, Karolinska Institutet-Stockholm University, Gävlegatan 16, 113 30 Stockholm, Sweden

## Abstract

Finding biomarkers constitutes a crucial step for early detection of Alzheimer's disease (AD). Brain imaging techniques have revealed structural alterations in the brain that may be phenotypic in preclinical AD. The most prominent polymorphism that has been associated with AD and related neural changes is the Apolipoprotein E (*APOE*) **ε**4. The translocase of outer mitochondrial membrane 40 (*TOMM40*), which is in linkage disequilibrium with *APOE*, has received increasing attention as a promising gene in AD. *TOMM40* also impacts brain areas vulnerable in AD, by downstream apoptotic processes that forego extracellular amyloid beta aggregation. The present paper aims to extend on the mitochondrial influence in AD pathogenesis and we propose a *TOMM40*-induced disconnection of the medial temporal lobe. Finally, we discuss the possibility of mitochondrial dysfunction being the earliest pathophysiological event in AD, which indeed is supported by recent findings.

## 1. Introduction

Alzheimer's Disease (AD) is one of the leading causes of dementia today and it poses an immense societal challenge as the prevalence is expected to continue to rise [1]. This makes it imperative to identify early preclinical changes in AD with high accuracy, in order for intervention strategies to yield effective outcome and to allow affected individuals to partake in an active treatment plan [2–5]. AD is characterized by early pathological changes in the brain, including senile plaques, neurofibrillary tangles, synapse, and neuronal loss. Neurofibrillary tangle formation may initiate in subcortical nuclei such as the dorsal raphe and locus coeruleus, prior to spreading to transentorhinal regions [6, 7]. Findings also support that pathological changes in AD commence in the medial temporal lobe (MTL) [8–10], primarily in the entorhinal cortex (ERC) and hippocampus (HC) [11–13], which undergo initial gray matter (GM) loss. Recently, attention has also been directed towards the impact of pathological mechanisms on white matter (WM), as up to 50% of AD cases present with global WM deterioration in neuropathological examinations [14, 15]. The temporal succession of GM and WM changes in preclinical AD remains to be determined; so far there is support for both primary and secondary WM changes within the MTL [16, 17].

**Figure 1:**
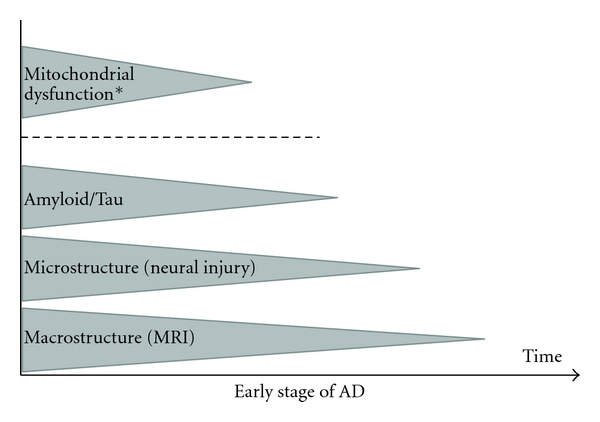
Timeline of potential biomarkers in Alzheimer's disease. Prior to clinical diagnosis of AD, beta-amyloid (A*β*) aggregation, micro- and macrostructural changes are thought to take place in a timewise fashion (adapted from [23]). *Recent research points to a shift in the biomarker timeline, with mitochondrial dysfunction being primary in the pathophysiological cascade of AD, eventually leading to micro- and macrostructural changes in the AD brain.

MCI is regarded as a prodromal state of AD, where individuals present with subjective memory complaints and/or objective memory impairment, but are still intact in daily life and do not meet current AD diagnostic criteria [2, 18, 19]. Amnestic type MCI (aMCI), where memory impairment is considered predominant, has been proposed as a solution for the diagnostic heterogeneity of the overall MCI criteria. The construct of MCI allows for the clinical assessment of prodromal AD, where early interventions could have a beneficial effect [20]. While promising, this therapeutic window is hampered by the fact that not all individuals with MCI convert to AD (6–25%), and almost half return to normal cognitive health within the first year of followup [2, 21]. Moreover, caveats remain regarding MCI and its clinical usefulness, signifying that the most beneficial use of the MCI criteria is by combination with other structural, functional, neuropsychological, genetic, and pathological biomarkers [22]. These biomarkers were recently placed into a hypothetic biomarker timeline by Jack and colleagues [23], who proposed that the pathological cascade in AD commences with amyloid and tau pathology, followed by neural injury and dysfunction and finally structural alterations (see Figure 1). Furthermore, they hypothesize that *β*-amyloid deposition and the following cascade occur earlier in Apolipoprotein (*APOE*) *ε*4 carriers. Recent findings have shown brain and cognitive changes up to 10 years prior to the diagnosis of AD, indicating that the combination of biomarkers may provide an alternative timeline [23–25]. A growing body of literature has emphasized the association between genetic and structural brain biomarkers, with imaging quantitative traits within the MTL being a more objective outcome than clinical diagnosis alone. The MTL may act as a mediator between genetic polymorphisms and the clinical expression of AD, indicating the advantage of combined genetic and brain integrity biomarkers [26, 27].

*APOE* is one of the primary AD polymorphisms, associated not only with risk and age of onset, but also brain integrity in AD [3, 28, 29]. Due to its Linkage Disequilibrium (LD) with *APOE*, Translocase of outer mitochondrial membrane 40 (*TOMM40*) was previously thought to have minimal influence on the risk of AD [30, 31]. Nevertheless, it is now established that *TOMM40 *influences onset of AD [31–37]. The *TOMM40* gene holds promising biomarker properties due to its negative impact on downstream apoptotic processes within the mitochondrial system via possible amyloid beta (A*β*) interplay [30, 38, 39]. Recently the mitochondrial cascade hypothesis has received increasing support, proposing that mitochondrial dysfunction is the key pathological mechanism in AD, influencing brain structures known to be vulnerable in AD [40, 41]. We intend to extend these theories by presenting the mitochondrial disconnection model, an adapted model for mitochondrial involvement in preclinical AD. We also suggest a timeline shift in the biomarker realm, away from the amyloid hypothesis, towards early and primary mitochondrial involvement in the pathophysiology of AD (see Figure 1). The implication of mitochondrial dysfunction in AD is currently supported by genetic and neuropathological research [30, 39, 41] and has the possibility to shed light on the primary biological insult in the disorder, as well as to provide a new therapeutic window for AD [42, 43].

The present paper focuses on recent advances in neuroimaging and genetic biomarkers for preclinical AD. After an overview of structural brain changes in early AD, we discuss the influence of *APOE* and *TOMM40*, in an effort to approximate the primary pathological cascade in AD. The mitochondrial disconnection model is an extension of previous findings and is suggested as a workable hypothesis from which the influential role of mitochondria on AD can be assessed.

## 2. Structural Brain Changes in Early Alzheimer's Disease

The consensus in the literature is that there is a long preclinical phase of AD, with cognitive as well as structural brain changes commencing years before clinical diagnosis of the disorder [23, 24, 44]. Indeed, significant brain atrophy can be observed in healthy individuals who will subsequently develop MCI or AD, in comparison to stable controls, within the bilateral medial and lateral temporal lobes, orbitofrontal cortex, posterior cingulate, and precuneus [24]. Interestingly, these preclinical changes correspond to the pattern of GM alterations seen in diagnosed AD [15, 45], demonstrating that early AD type pathology is present prior to clinical symptoms [24, 46].

Both MCI and AD have characteristic influence on structures in the brain, thereby making them dissociable from nonpathological aging [45, 47]. Imaging studies have been able to confirm the Braak staging of neuropathology in AD by showing early structural changes within the MTL (more particularly the ERC and HC), prior to spreading to adjacent cortices [10, 13, 48–50]. Looking closer at the HC, the lateral CA1 subfield is the most vulnerable in MCI and AD while GM loss in the subiculum is associated with nonpathological age-related changes, denoting region-specific changes within the HC in AD [51]. Moreover, several studies have indicated that the rate of atrophy within the MTL is faster for those who convert from normal aging to MCI as well as from MCI to AD in comparison to those who remain stable [52–54]. This shows that not only atrophy, but also the rate of atrophy in the HC over time could serve as a potential biomarker of preclinical AD. Structural changes in mild-to-moderate AD also occur in areas that are strongly connected to the MTL, such as the retrosplenial cortex, posterior cingulate, precuneus, and lateral posterior parietal regions [55, 56]. These findings have been confirmed in individuals with MCI, where medial and lateral temporal as well as parietal atrophy was evident in individuals who converted from MCI to AD, in comparison with those who remained stable [57].

Although the progression of WM alterations in the brain is still unclear, damage to WM pathways within the MTL can be detrimental according to the “disconnection hypothesis,” stating that deterioration of WM tracts leads to subsequent disconnection of the brain's circuitry [11, 58]. Not only might there be an overall disconnection, but a specific isolation of the HC that may result from reduced WM integrity within the MTL, mainly in the parahippocampal area, cingulum, fornix, uncinate fasciculus, and perforant pathway [59–61]. Moreover, an alteration in the WM of the precuneus, closely interconnected with the MTL, has also been observed, resulting in the isolation of the hippocampus [59]. Researchers debate the sequential order of GM and WM deterioration in preclinical AD and the Wallerian degeneration hypothesis stipulates that loss of WM integrity is secondary to GM changes. In line with this hypothesis, research has shown GM atrophy to be more efficient in distinguishing between AD patients and healthy controls. More specifically it has been observed that right-sided hippocampal GM loss is a better predictor of diagnostic status of AD than measures of WM integrity [8]. Recently, this hypothesis was supported in a study where primary GM degeneration in the HC was followed by Wallerian degeneration of WM within the interammonic commissure, a pathway connecting the left and right hippocampi [12]. Also similarly, Villain and colleagues found HC atrophy to be followed by loss of WM integrity in the uncinate fasciculus and cingulum bundle, which was corroborated by metabolic alterations in connected cortical areas, demonstrating a significant disruption in connectivity [61]. By contrast, WM deterioration has also been observed in the absence of primary GM changes [60, 62]. For example, in individuals with MCI and AD, loss of WM integrity has been seen in the perforant pathway in absence of GM atrophy [63]. Alteration of the perforant pathway, which connects the ERC and the HC and constitutes a gateway to the limbic system, may contribute to early and likely initial disconnection of the MTL in preclinical AD [60].

WM changes in small pathways of the brain, such as the perforant pathway, are still arduous to discern with the current available neuroimaging techniques. This is particularly important in preclinical AD where the areas implicated are small WM pathways within the MTL. Moreover, the lack of longitudinal studies renders it difficult to determine the temporal order of GM and WM changes in the brain. It has been pointed out that it is disadvantageous to consider the WM changes in the brain in a dichotomized fashion [16, 17]. Instead, a balanced view has been proposed where the temporal order of structural changes in the brain is dependent on the retrogenesis of the specific structure [16, 64]. For instance, in late myelinating pathways connected to the MTL such as the inferior longitudinal fasciculus, primary loss of WM integrity is thought to be of major influence. In early myelinating pathways such as the cerebral peduncles, posterior limb of internal capsule, and forceps major on the other hand, WM degeneration is considered secondary to GM loss. However, it has been proposed that within each brain area, depending on its retrogenetic development, there is a ratio between primary and secondary WM degeneration, possibly explaining why the temporal order of GM and WM changes in early AD has been difficult to ascertain [16].

## 3. *APOE* and Preclinical Alzheimer's Disease

While research in the genetic field has been fraught by small effect sizes and difficulty in replicating findings, *APOE* has remained a robustly replicated susceptibility gene for AD [42, 65, 66]. Located on chromosome 19, *APOE* translates into three common allelic variations *ε*2, *ε*3, and *ε*4 [67], the *ε*4 being strongly associated with risk of developing AD [28, 68, 69]. Furthermore, the *ε*4 allele has been associated with decreased memory functioning, processing speed, and loss of GM and WM integrity [70–74]. The *ε*4 allele also modulates risk of progression from MCI to AD. In effect, a recent meta-analysis demonstrated that the presence of one or two *ε*4 alleles increased the risk of MCI conversion to AD up to four times. However, *APOE* as a risk factor has low predictivity and sensitivity values as a diagnostic test for AD, leading to the conclusion that *APOE* genotyping has limited value as a diagnostic tool in clinical practice [75–77].

 APOE appears to play an essential role for lipid metabolism within the Central Nervous System (CNS) and allelic variations of the gene are thought to modulate neural repair, lipid homeostasis, oxidative stress, and A*β* deposition [43, 68]. As lipids are abundant in the brain and essential for myelination of axons, it comes as no surprise that APOE, being the main cholesterol transport lipoprotein, appears to play an essential role in maintaining brain integrity [78]. Although the mechanism behind the influence of APOE on the brain is not fully elucidated, the protein appears to govern the efficiency of cholesterol delivery to neurons. Particularly, the presence of an *ε*4 allele reduces the delivery of cholesterol, consequently disturbing lipid homeostasis within the CNS and triggering a cascade leading to the formation of amyloid depositions [79]. The combined amyloid cascade hypothesis [80] and APOE lipid recycling cascade models [81] promote a disturbance in lipid homeostasis as a source for AD pathology [82]. While the amyloid cascade hypothesis has been prominent throughout the last two decades, it was initially based on studies with rare autosomal dominant variants of AD and had pathophysiological shortcomings [83]. Indeed, widespread amyloid deposition is present in AD, but there is no consensus regarding the finite pathophysiological burden of amyloid in the brain and it has been argued that amyloid aggregation is a downstream process in AD not related to clinical manifestation of the disorder [84]. While *APOE* may influence the amyloid cascade in AD, other neuropathological aspects of the polymorphism have been highlighted, including influence on neuronal repair mechanisms and maintenance of synaptic connections [43]. One way of increasing the predictability of the *APOE* polymorphisms is by combining genetic and structural brain biomarkers [27, 73].

## 4. *APOE* and Structural Integrity

Extensive research has been done on the genetic influence of *APOE* polymorphisms on brain changes in preclinical AD (Table 1). Support for the influence of *APOE* on AD-like changes within the brain comes from a Genomewide Association Study (GWAS) on neuroimaging phenotypes in a mixed sample of MCI and AD individuals [85]. The authors found *APOE* to be one of the top ten genetic markers to influence overall imaging phenotypes. Espeseth and colleagues [86] demonstrated a modulatory effect of *APOE* polymorphism on cortical thickness in healthy middle-aged adults. Carriers of an *ε*4 allele showed accelerated cortical thinning in specific regions known to structurally deteriorate in normal aging but also in AD such as prefrontal regions, parahippocampal cortex, and adjacent occipitotemporal areas (fusiform and lingual gyri), but not the HC. Others have found more region-specific influence of *APOE* on MTL areas [29, 87, 88]. This effect appears to be left lateralized with the *ε*4 influence on HC volume [89]. The majority of findings converge towards a significant impact of *APOE* polymorphism on GM integrity within the MTL, mainly the HC.

 Further, evidence for the influence of *APOE* on the MTL comes from longitudinal studies on MCI and conversion to AD (see Table 1). These have found that there is a genetic influence of *APOE* not only on hippocampal GM loss, but also on the rate of atrophy of the HC [88, 94]. Support for the specific influence of *APOEε*4 in MCI has been shown, as aMCI individuals have been found more likely to have smaller hippocampi and be carriers of at least one *ε*4 allele than nonamnestic MCI individuals [94]. Also, MCI *APOEε*4 carriers express AD-type structural alterations such as atrophy in MTL regions (ERC and HC). Those with MCI and an *ε*4 allele who convert to AD also show atrophy in frontal and parietal cortices [48, 95]. Progressive MCI *ε*4 carriers show global AD-type structural changes years before clinical diagnosis of AD [95]. However,* APOE ε*4 does not predict conversion from MCI to AD, while ERC volume reduction at baseline does [48]. Hence, it appears that while *APOE* may influence structural integrity in areas that are vulnerable in the preclinical stages in AD, *APOE* polymorphism has limited predictive value on the conversion to AD. The latter finding may, however, be biased by limited sample sizes. Thus, future studies combining structural, genetic and cognitive biomarkers in larger samples may show enhanced predictability.

Given its hypothesized role as the brain's main lipid transporter [79], *APOE* impacts WM integrity in preclinical AD [99]. Several studies have confirmed both widespread and localized WM changes throughout the brain in relation to *APOE* polymorphism in healthy samples (see Table 1). Persson and colleagues [97], for instance, demonstrated an impact of *APOEε*4 on the WM integrity of the posterior corpus callosum and HC in healthy younger and older individuals, possibly reflecting preclinical signs of AD. Their findings are supported by recently published data showing that the presence of an *ε*4 allele exacerbates age-related WM changes [73]. Moreover, it seems that late myelinating regions are more susceptible to age-related loss of integrity in *ε*4 carriers, leading to progressive disconnection of the brain in *APOE ε*4 carriers [96].

In conclusion, influence of *APOE* on GM structural integrity has been consistently demonstrated in areas associated with preclinical AD. By contrast, little is known about the genetic influence of *APOE* on WM changes in AD and whether these changes are occurring sequentially or in a balanced retrogenetic fashion.

## 5. *TOMM40* and Preclinical Alzheimer's Disease

Missing heritability is increasingly debated in the literature, as current genetic findings are not able to explain the full extent of the genetic contribution to complex diseases such as AD [65, 100, 101]. While larger sample sizes in GWAS are suggested as a remedy for missing heritability, others suggest that the answer resides in genetic polymorphisms that are in LD with current known ones [65, 102–104]. *TOMM40* is becoming increasingly acknowledged as a prominent AD gene [31–37]. In LD with *APOE*, *TOMM40* could hold part of the missing heritability that we are searching for in our efforts to map the genetic influences in AD. Moreover, taking *TOMM40 *into consideration may contribute to a better understanding of the early and primary pathophysiological cascade that takes place in the preclinical phases of the disorder. This hypothesis is supported by the fact that *TOMM40* asserts its influence on mitochondrial survival, a process increasingly highlighted in the pathogenesis of AD [31, 105, 106]. Mitochondrial dysfunction has been associated with several pathological processes in AD, including brain hypometabolism, synaptic pathology, accumulation of Amyloid Precursor Proteins (APP), and A*β* influx to the cell [38, 39, 41]. Mitochondria have recently been implicated in more complex signaling cascades, oxidative stress, and apoptotic processes, indicating that mitochondria are not merely a powerhouse of the cell, rather they appear to govern cell death [107]. The notion of mitochondrial dysfunction in aging and neurodegeneration is not new. In fact, malfunctioning mitochondrial systems have been observed in premature aging [105, 108] as well as neurodegenerative disorders such as AD [40, 109, 110], Parkinson's and Huntington's disease [105] and appear to have an early and causal influence on pathological processes in the brain. Damage in mitochondria may exert a specific influence on the pathophysiology of AD through interplay with A*β* and its precursor, the APP [111].

 Mitochondria play an essential role in providing energy to cells and are abundant in the neurons and synapses of the CNS. Containing an outer and inner membrane, the organelle is essential for the production of adenosine triphosphate (ATP), which is the energy source of all cells [112]. The outer mitochondrial membrane contains the translocase of outer mitochondrial membrane pore subunit (Tom40). The Tom40 channel forming subunit is one of the primary pores via which proteins can readily enter the mitochondria. The pore is governed by the *TOMM40* gene and is essential for mitochondrial survival as the majority of proteins that enter the mitochondria pass through here [107, 113]. In AD specifically, it has been hypothesized that mitochondria exert neurotoxic influence by allowing the influx of A*β* to the cell via the Tom40 import pore. Passage of A*β* through the Tom40 import pore increases Reactive Oxygen Species (ROS) within the organelle. This increase is detrimental for mitochondrial survival and energy production (ATP), ultimately resulting in apoptotic processes of the cell [38, 111, 114]. Further ROS precipitating events include the accumulation of APP in mitochondrial import pores. This accumulation of APP in import pores has been found in AD brains, mainly in the frontal cortex, HC, and amygdala and seen to vary with disease severity. Intriguingly, *APOEε*3/*ε*4 carriers endorse the highest amount of mitochondrial APP, suggestive of a synergetic effect of mitochondrial dysfunction in the presence of *APOE* [39]. Furthermore, it has been shown that mitochondria have high intracellular A*β* accumulation in AD [114]. It has been pointed out that A*β* accumulation in mitochondria precedes extracellular A*β* deposition, which supports the role of mitochondria in the pathogenesis of AD [38]. Moreover, *TOMM40* has recently been associated with CSF biomarkers including A*β*_1-42_, t-tau, and p-tau [115]. To this end, the mitochondrial cascade hypothesis is receiving increasing support throughout the literature, thereby demonstrating the implications of mitochondrial dynamics in the early pathophysiology of AD. The hypothesis postulates that mitochondrial dysfunction precedes amyloid insult to the brain and that mitochondrial injury is the primary source of pathology in AD [40, 116].

A recent neuropathological study, investigating the morphology of mitochondria in AD brains, confirmed the presence of mitochondrial pathology in brain areas typically associated with AD-type pathology [41]. Here mitochondrial alterations of shape and size were observed in AD, in comparison to healthy controls, in the neurons of the HC, neocortex, cerebellum, thalamus, pallidum, red nucleus, and locus coeruleus. As these assessments were conducted in postmortem AD brains, they more likely represent late pathophysiological changes in AD. However, these findings are suggestive of morphological changes in mitochondria, possibly acting causally in the pathogenesis of AD. While mitochondrial morphological changes were not limited to the HC [41], one would expect to see preclinical morphological changes in the MTL, based on previous findings of mitochondrial- induced oxidative stress in preclinical dementia [110] as well as findings of A*β* and mitochondrial interplay [114].

 Further support for mitochondrial involvement in AD comes from genetic studies involving the *TOMM40* gene. Primarily Roses, and colleagues, the same group that discovered the influence of *APOE* on AD [28], have been able to demonstrate an association between a long poly-T repeat of the *TOMM40* gene with earlier age of onset of AD in *APOEε*3 carriers [30]. The *TOMM40* poly-T length acts either dependently or independently of *APOE* in the pathophysiology of AD [117]. Moreover, studies focusing on Single-Nucleotide Polymorphisms (SNPs) have found an association between *TOMM40* and AD. A recent case-control study, comparing individuals with or without AD, showed a highly significant relationship between a *TOMM40* SNP (rs2075650) and AD. Interestingly, a haplotype of *TOMM40* rs2075650, rs11556505, and *APOE* rs429358 held a stronger association with AD than *TOMM40* rs2075650 alone [85], supporting Roses and colleagues findings of a synergetic effect of *TOMM40* and *APOE* [30]. Moreover, a recent genetic association study suggested that protein transport across the mitochondrial membrane was implicated in the pathophysiology of AD, and that *TOMM40* is a likely contributor to this detrimental transmembrane process within the mitochondria [118].

 Although genetic studies on mitochondrial involvement in AD are in their initial stages and replications are warranted, findings are supportive of previous postmortem, animal, and pathological studies in AD suggesting a significant involvement of mitochondrial dysfunction in AD.

## 6. *TOMM40* and Structural Integrity

Postmortem studies on mitochondrial morphology in the HC [41] and the presence of APP in mitochondrial import pores in the HC of AD patients [39] suggest that mitochondrial dysfunction may follow Braak staging of neuropathology [8], with degeneration commencing in the MTL. By assessing the mitochondrial influence on brain integrity in AD, this temporal association can be further evaluated. Current cross-sectional studies, focusing on the differential influence of *TOMM40* polymorphisms on the brain, offer promising insight to this link between genes and neuropathology in AD.

 Johnson and colleagues [98] assessed the influence of *TOMM40* poly-T length on structural brain integrity and cognition among *APOEε*3 carriers. Analyses were restricted to areas known to be vulnerable in AD including the amygdala, HC, parahippocampal gyrus, posterior cingulated, and precuneus. *APOEε*3 carriers were divided according to length variations of the *TOMM40* polymorphisms, homozygous short (SS), homozygous very long (VL), and heterozygotes (S/VL). *TOMM40* length variation was found to influence episodic memory, which strongly depends on HC integrity, exemplifying the genetic involvement of *TOMM40* on AD-type cognitive deficits. On the brain level, the poly-T length seems to influence the integrity of the medial ventral precuneus and posterior cingulate [98], which have been shown to be the site of early amyloid burden in AD [55]. This confirms previous findings, where the influence of *TOMM40* poly-T length on AD onset has been shown [31] and supports the notion of mitochondrial influence in areas of the brain that are vulnerable to AD. Hence, it appears that healthy middle-aged individuals, who are *APOEε*3 homozygotes with a long poly-T of the *TOMM40* gene, show an AD-like profile with regards to cognitive performance as well as structural brain changes.

 GWAS with HC volume as the phenotype supported the influence of the *TOMM40* gene on structural integrity of areas implicated in AD. The authors found that three *TOMM40* risk alleles (rs157580, rs2075650, and rs11556505) were overrepresented in the AD population as assessed by case-control analysis [27]. Shortcomings of focusing on one region in the brain were overturned, and a recent GWAS used whole brain imaging phenotypes in an attempt to understand the association between *TOMM40* and structural integrity [85]. Notably, this analysis resulted in a significant association of the *TOMM40* gene (rs2075650) with left amygdala and bilateral HC volume. Furthermore, comparison of healthy versus AD individuals revealed that *TOMM40* was among the top 5 SNPs associated with whole brain imaging phenotypes. This points to the selective influence of *TOMM40* on structural integrity in brain areas vulnerable to AD and supports previous findings of high APP burden in mitochondrial import pores in the HC and amygdala [85].

 In an effort to examine the influence of *TOMM40* in an independent cohort, we used data from nondemented individuals (age range: 60–90 years), from the Swedish National study on Aging and Care in Kungsholmen (SNAC-K) [119]. We assessed the genetic influence of the *TOMM40* (rs2075650) gene on GM volume of the HC and episodic memory performance [120]. We expected to observe an *APOE*-independent negative influence of *TOMM40* G (risk allele) on both cognitive performance and volume. Based on previous studies where *APOE*-independent *TOMM40* influence was assessed [117], we stratified our *TOMM40* sample across *APOE*. While we found no independent effect of *TOMM40* on HC or ERC volume per se, we did observe that the positive association between HC volume and episodic memory was driven by the presence of at least one *TOMM40* G allele in *APOEε*4 carriers. This finding indicates that carriers of a *TOMM40* G allele may be more dependent on HC volume for accurate episodic memory performance. This study suggests alterations within the mitochondrial system in *TOMM40* G allele carriers, perhaps resulting in early morphometric alterations in mitochondrial shape and size. These alterations are not influencing structure, but rather the function of the HC, as assessed by episodic memory performance. It is possible that we are observing functional alterations at an early stage that are not yet accompanied by significant volumetric changes in aging. As the timeline shifts to neurodegeneration, these functional changes may result in substantial structural changes, supported by postmortem findings of morphometric alterations in the mitochondria of the HC in AD [41]. Further support is provided by *TOMM40* influence on brain integrity and cognition that are vulnerable in MCI and AD, as well as the overrepresentation of *TOMM40* risk alleles in AD population [27, 98]. Functional changes within the HC might therefore be a primary sign of mitochondrial degeneration in preclinical AD.

Overall, the studies that are available today point to a selective influence of *TOMM40* polymorphisms on structural changes in AD vulnerable areas such as the HC, precuneus and posterior cingulate cortex. To our knowledge, no studies have been conducted on the genetic influence of *TOMM40* on WM changes in the brain. While the majority of findings concerning *TOMM40* implicate GM changes, recent findings from our laboratory suggest that mitochondrial dysfunction might influence hippocampal functioning as well, as assessed using cognitive testing. These findings are supportive of a prominent mitochondrial dysfunction in AD and are promising for the utilization of mitochondrial biomarkers for the accuracy of early detection of preclinical AD.

## 7. The Mitochondrial Disconnection Model

**Figure 2:**
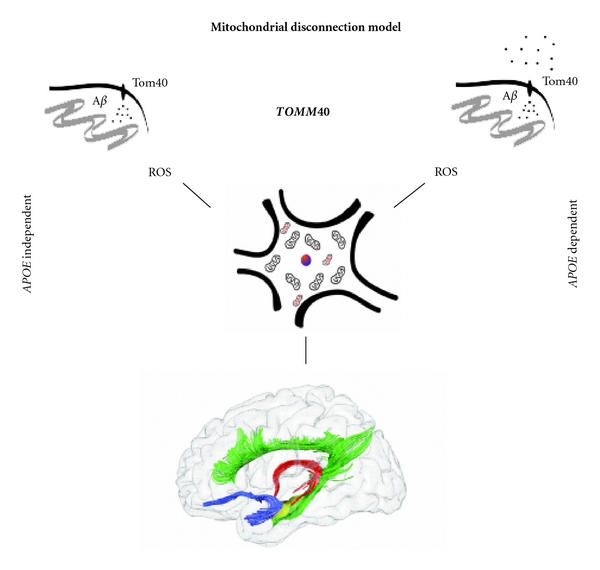
The mitochondrial disconnection model is an extension of the *TOMM40*-induced mitochondrial cascade in Alzheimer's disease (adapted from [31, 117]). *TOMM40* governs the Tom40 complex on the outer mitochondrial membrane, allowing the influx of amyloid beta (A*β*) into the organelle. *TOMM40* influence occurs either independently or dependently of *APOE*. Nevertheless, *TOMM40*-induced influx of A*β* to the cell starts downstream apoptotic processes via Reactive Oxygen Species (ROS), inducing cell death. We hypothesize that this results in early functional and structural alterations within the Medial Temporal Lobe (MTL), primarily in the hippocampus (yellow). Subsequent disconnection of the MTL, via deterioration of White Matter pathways such as the cingulum (green), fornix (red), and uncinate fasciculus (blue) follow. Disconnection of the MTL may induce secondary functional and structural alteration in distal areas possibly as a result of primary mitochondrial-induced cell death. (Brain graphic: courtesy of Michel Thiebaut de Schotten from the Natbrainlab, King's College, London, UK.).

As an attempt to recapitulate and expand on findings in the field, we propose the mitochondrial disconnection model (see Figure 2). This model is an adapted representation of the mitochondrial cascade in AD, and its downstream influence on structural brain changes. We propose that this cascade has a primary influence on GM structural integrity of regions of the MTL, leading to disconnection and isolation of the MTL as a result of deterioration of connecting WM tracts.

In the adapted mitochondrial disconnection model *TOMM40* acts via *APOE*-independent and -dependent pathways [31, 117]. Via *APOE*-independent pathways, *TOMM40* regulates A*β* influx to the mitochondria via the Tom40 outer membrane pore. This notion is in line with postmortem studies that have found APP lodged in the Tom40 channels [39] as well as genetic studies suggesting that protein transport across the mitochondrial membrane, that is governed by the *TOMM40* gene, is implicated in the pathophysiology of AD [118]. Via *APOE*-dependent pathways, there may be an interaction between *APOE* and *TOMM40*, which in turn may influence the A*β* influx. *APOE* is essential in the clearance and deposition of A*β* [79, 121–123] and has also been shown to increase extracellular A*β* availability [39, 43]. This increase in *APOE*-induced A*β* availability may allow for a larger proportion of A*β* to flow into the mitochondria via Tom40 import pores [117]. *APOEε*3/*ε*4 carriers have the highest amount of mitochondrial APP, resulting in impaired mitochondrial functioning, suggestive of the importance of mitochondrial dynamics, even in *APOEε*3/*ε*4 carriers [39]. There is support in the literature for both *APOE*-independent and -dependent pathways. Moreover, the *TOMM40*-induced mitochondrial cascade is unlikely to be autonomous of *APOE*, considering that *APOE* and *TOMM40* are genetically linked via LD. Nevertheless, we propose that even in the *APOE*-dependent pathway, the role of *TOMM40 *is primary, as it influences mitochondrial protein transport via the Tom40 import pore.

Irrespective of the pathway through which the mitochondrial cascade commences, the flow of A*β* into the organelle induces apoptotic processes. The latter functions by increasing ROS within the mitochondria and has detrimental effects on cell survival within the MTL. As neurons contain hundreds of mitochondria, apoptotic processes may occur in a gradient fashion and might not influence neuronal structure initially. Morphometric changes have been seen in the mitochondria of the HC in AD, but these take place in the later stages of the disorder [41]. It is possible that early *TOMM40*-induced mitochondrial changes likely influence HC function, rather than its volume, evidenced by the triad between *TOMM40*, HC volume, and episodic memory in our aforementioned ongoing study [120]. This points to the importance of combining genetic, structural, and cognitive biomarkers to assess preclinical AD, as structural brain changes alone might not be sufficient for early and accurate prediction of preclinical mitochondrial alterations in AD. We suggest that functional changes in the HC, as a result of early mitochondrial alterations, could be utilized as an additional biomarker for preclinical AD. A timeline of mitochondrial degeneration commencing with early functional changes followed by structural changes within the brain can be hypothesized.

Nevertheless, initial *TOMM40*-governed mitochondrial insult has been found to take place in the HC [85]. Based on previous findings that disconnection and isolation of the HC plays an important role in the early pathophysiology of AD [59–61], we hypothesize that WM changes of directly HC-connected WM tracts including the fornix, cingulum, and uncinate fasciculus follow in the mitochondrial cascade (see Figure 2). Whether this occurs via primary Wallerian degeneration or balanced retrogenesis remains to be elucidated. WM changes have been shown to be both primary and secondary to GM alterations in AD. The balance between primary or secondary WM degeneration within a certain region may be dependent on the gradient of mitochondrial dysfunction within that area. This balance might not be the same throughout the brain, as mitochondrial dysfunction has been primarily observed in MTL structures such as the HC and amygdala [39]. Future longitudinal studies will have to discern the temporal order of events and whether WM changes in preclinical AD are dependent on mitochondrial dysfunction in the GM. Overall, a succession of mitochondrial dysfunctional events in the pathophysiology of AD is supported not only by our ongoing study, but also by studies showing mitochondrial damage in normal aging [124, 125]. The degree of how widespread mitochondrial injury is in the brain may be determined by the neurodegenerative status of the individual and may follow Braak staging of pathology. That would explain why we observe *TOMM40*-induced influence on structural integrity of areas implicated in the early stages of AD. Based on these findings, we propose that widespread GM atrophy, seen in the later stages of AD, results from mitochondria-induced MTL disconnection via cortico-limbic pathways. Disconnection of the MTL may induce secondary functional and structural alteration in distal areas [61].

 The proposed model is a representation of mitochondria-induced disconnection as an early and accurate biomarker for preclinical AD (see Figure 1). By this we expand on Jack and colleagues [23] dynamic biomarker timeline and propose that mitochondrial dysfunction initiates the pathophysiological cascade in AD. Findings in support of this timeline include the selective influence of *TOMM40* on AD onset, HC volume, and cognition over and beyond that of *APOE* alone [27, 31, 85]. Moreover, mitochondrial A*β* aggregation precedes extracellular A*β* aggregation [38] supporting the mitochondrial cascade hypothesis rather than the amyloid cascade hypothesis, as the primary event in the biomarker timeline of AD. However, Jack and colleagues [23] pointed out that A*β* depositions are also observed in asymptomatic individuals, suggesting that the amyloid pathological process might be part of the process of aging. While amyloid depositions precede the clinical outcome of AD, the high presence of A*β* in healthy individuals suggests that other factors are at play. Moreover, the amyloid cascade does not fall into line with the Braak staging of pathology, where tau pathology was proposed to precede amyloid aggregation and commence within more basal midbrain structures [6, 7]. It has been shown that both tau and amyloid have synergic effects on mitochondrial dysfunction [126], suggesting that a biomarker timeline based on mitochondrial pathology might be more accurate in AD and would reconcile with Braak staging of pathology that has been well supported by neuroimaging studies.

## 8. Conclusion

There is increasing evidence for a primary mitochondrial involvement in the pathophysiology of AD, as mitochondria have been found to regulate cell death. Several studies have highlighted the importance of altered mitochondrial dynamics in the preclinical stages of AD, as well as mitochondrial involvement in structural brain changes within the MTL. Perhaps more importantly, mitochondrial dysfunction appears to be primary to extracellular A*β* aggregation. These findings demonstrate the necessity to direct attention away from the amyloid cascade hypothesis towards the mitochondrial cascade hypothesis. Moreover *TOMM40 *should be considered as a possible genetic modifier of the biomarker timeline in AD. The distinction between amyloid and mitochondrial cascades is not arbitrary with consideration to potential biomarkers and treatments of AD. While “mitochondrial protectors” as a potential treatment for AD are currently under investigation [116], further studies are needed in order to assess mitochondrial dynamics in preclinical AD. Genetic polymorphisms such as *TOMM40* have the potential not only to assess individuals at risk, but also to serve as biomarkers in combination with current known structural and cognitive ones. We propose the mitochondrial disconnection model as a means by which the mitochondrial dynamics can be assessed in preclinical AD.

## Figures and Tables

**Table 1:** Genetic influence of *APOE* and *TOMM40* on cerebral structural integrity.

Author	Population	Method	Structural integrity	Conclusion
			*Alzheimer's Disease*	

			***APOE***	
Pievani et al. 2011 [29]	Across *APOE* (*ε*4) *n* = 28	Volumetry region based	Smaller HC in *APOEε*4+	*ε*4+ carriers have greater atrophy in the HC.

Bendlin et al. 2010 [90]	Across *APOE*(*ε*4) & family history of AD*n* = 136	DTI whole brain	*Parental history of AD * Reduced FA in cingulum, tapetum, uncinate fasciculus, HC, and adjacent WM No main effect of *APOE* on WM, but interaction with family history where family history and *ε*4+ induced reduced FA	While no main effect of *APOE* was observed onDTI measures, parental history of AD was associated with reduced WM integrity in brain areas deteriorated in AD, which in turn interacted with *APOE*.

Pievani et al. 2009 [91]	Across *APOE* (*ε*4) *n* = 29	Volumetry whole brain	*APOE *ε*4+ * Significant atrophy in Bil temporal lobes, occipital lobes, retrosplenial, and posterior cingulate Highest GM reduction >20%: entorhinal cortex, anterior temporal pole, superior and middle temporal gyrus, ventral, and dorsal occipital cortex *APOE *ε*4+ versus *ε*4*− Global GM reduction comparable (RH: 14 versus 15%; LH: 16 versus 17%) *ε*4+ more atrophy in medial and lateral temporal lobes, and right occipital pole	After assessing the whole cortical mantle, greater susceptibility of the MTL area was found in *APOEε*4 carriers.

Filippini et al. 2009 [92]	Across *APOEε*4 *n* = 100	Volumetry whole brain	*Additive model * GM reduction in Bil MTL (HC, amygdala, parahippocampal gyrus), fusiform cortex, and orbitofrontal cortex *Genotypic model * Partially overlapping with additive, extending from posterior MTL to inferior lateral temporal cortex	Dose-dependent decrease in medial and anterior temporal lobe volume per allelic (*ε*4) load. Variable regional association indicating that *APOE* works differently on mechanisms of disease expression.

Barber et al. 1999 [93]	AD across *APOEε*4*n* = 25	Visual scoring MTL atrophy WM HI	No significant differences between *ε*4+ and *ε*4− on MTL atrophy, WM HI	*APOE* does not modulate white and gray matter in AD. While *APOE* influences risk of AD it appears not to modulate pathological processes after diagnosis.

			***TOMM40***	
Potkin et al. 2009 [27]	AD (*n* = 229) Healthy Controls (*n* = 194)	Volumetry region-based GWAS on HC QT	Case-control analysis identified *APOE* and a new risk gene *TOMM40* at 10^−6^ (10^−11^ at a haplotype level between *APOE* & *TOMM40* rs11556505) 25 SNPs were associated with QT HC, including *APOE *	*APOE* has an effect on brain atrophy independent from overrepresentation in AD. A novel risk gene, *TOMM40*, was found to be associated with AD.

			*Mild Cognitive Impairment*	

			***APOE***	
Spampinato et al. 2011 [88]	Stable versus Progressive MCI (*n* = 55) across *APOE (*ε*4) *	Volumetry whole brain Longitudinal	*Progressive APOE*ε*4+ * 1 year prior to diagnosis: GM atrophy in right temporal lobe, HC, insula 1 year FU: GM atrophy Bil HC, parietal, insula, caudate *Stable APOE*ε*4+ * 1 year FU GM atrophy Bil insula, temporal lobe	*APOE*ε*4+* converters show early GM loss 1 year prior to diagnosis, and atrophy progresses in *ε*4+ converters to AD. However, some MTL atrophy is present in *APOEε*4+ nonconverters, reflecting nonlinear effects of *APOE *ε*4*.

He et al. 2009 [94]	MCI across *APOE n* = 153	Volumetry region based Cross-sectional	*Amnestic MCI * Significantly reduced HC volume	Amnestic MCI individuals are more likely to have MTL atrophy and to be carriers of an *APOE *ε*4* allele.

Tapiola et al. 2008 [48]	Stable versus Progressive MCI across *APOE n* = 60	Volumetry region based Longitudinal	*Progressive APOE *ε*4+ * Reduced HC and ERC volume	While significant atrophy was seen within the MTL in *APOE *ε*4+* carriers with progressive MCI, the presence of an *ε*4 allele did not predict conversion to AD.

Hamalainen et al. 2008 [95]	Stable versus Progressive MCI (*n* = 56) across *APOE* (*ε*4)	Volumetry whole brain Longitudinal	*Progressive APOE *ε*4+ * Atrophy left inferior frontal gyrus, intraparietal sulcus *Stable APOE *ε*4+ * Atrophy right amygdala, anterior HC	*APOE *ε*4+* converters display global AD-like atrophy in frontal and parietal cortices in comparison to *ε*4−, 2.5 years prior to diagnosis of MCI.

			***APOE & TOMM40***	
Shen et al. 2010 [85]	*APOE* *n* = 818	Volumetry whole brain GWAS Freesurfer QT: 56 areas VBM QT: 86 areas	*APOE* rs 429358 (*ε*4 dependence) associated with whole brain Freesurfer (15 regions) and VBM (4) phenotypes at 10^.6^ significance *TOMM40* rs2075650 associated with Freesurfer (5) at 10^−7^ significance *Freesurfer phenotypes * *APOE* associated with widespread phenotypes *TOMM40* specifically associated with left and right hippocampi and left amygdala	While *APOE* is associated with widespread cortical AD-like changes, *TOMM40* appears to be associated mainly with MTL phenotypes. Both *APOE* and *TOMM40* were found among the top 5 SNPs in the GWAS.

			*Normal Aging (only cross sectional)*	

			***APOE***	
Ryan et al. 2011 [73]	*APOE n* = 126Age range52–92	DTI region based	Significant differences in ADC and FA with increasing age in frontal WM, lateral parietal WM, centrum semiovale, genu and splenium of CC, temporal stem WM These age-related differences in WM integrity were more prominent in *ε*4+	*APOE *ε*4* exacerbates age-related WM changes.

Zhang et al. 2011 [89]	*APOE n* = 409Age range70–90	Volumetry whole brain/region based	Reduced GM volume in left HC in *APOE *ε*4+ * No significant differences in basal forebrain	Only left hippocampal volume was significantly reduced in *APOE *ε*4* carriers and no differences were observed in the basal forebrain area.

Espeseth et al. 2008 [86]	*APOE ε*4+ (*n* = 37)*ε*4− (*n* = 59)Age range 48–75	Volumetry whole brain	No group differences in total brain volume, GM volume, WM volume *Cortical thickness *ε*4+ * Thicker cortex in bilateral occipital and occipito temporal areas, right parahippocampal gyrus and frontal areas	Thicker cortex in *APOE *ε*4+* was found in regions adjacent to those that show accelerated age-related decline, indicating that although well preserved now they may eventually show cortical thinning.
			*Age related cortical thickness *ε*4+ * Both *ε*4+ and *ε*4− have age-related thinning in occipital and insula, but *ε*4+ also show thinning of MTL	*APOE *ε*4* may accelerate thinning in areas that decline with aging (medial prefrontal, pericentral cortex) as well as areas susceptible to A*β* aggregation (occipitotemporal, temporal cortex).

Bartzokis et al. 2006 [96]	*APOE n* = 104Age range 55–75	DTI region based	*APOE *ε*4+* showed steeper age-related decline in radial diffusivity in late myelinated regions frontal lobe and genu of the CC	Late myelinated frontal regions appear more susceptible to age-related breakdown in *APOE *ε*4+* carriers. This leads to progressive disconnection of cerebral networks in *ε*4 carriers and is supportive of an anterior-posterior WM degeneration gradient.

Persson et al. 2006 [97]	*APOE n* = 60Age range 49–79	DTI region based	*APOE *ε*4+* show reduced FA in posterior CC, frontal fasciculus and HC	Supportive of previous findings of reduced FA in posterior CC, an area thought to be associated with AD pathology.

			***TOMM40***	
Johnson et al. 2010 [98]	*TOMM40* across *APOEε*3*n* = 117 Age range 40–65	Volumetry whole brain	Dose-dependent increase in *TOMM40* poly-T length associated with reduced GM volume in ventral posterior cingulate and medial ventral precuneus	A subgroup of *APOE *ε*3* carriers with long poly-T length of the *TOMM40* gene show brain changes in areas associated with AD. This indicates independent influence of *TOMM40*.

ADC: Apparent diffusion coefficient; *APOE*: Apolipoprotein E; Bil: Bilateral; CC: Corpus Callosum; DTI: Diffusion Tensor Imaging; ERC: Entorhinal cortex; FA: Fractional Anisotropy; FU; Follow up; GWAS: Genome Wide Association Studies; GM: Gray matter; HC: Hippocampus; HI: Hyperintensities: LH: Left Hemisphere; MD: Mean Diffusivity; MTL: Medial Temporal Lobe; QT: Quantitative Trait; RH: Right hemisphere; SNP: Single Nucleotide Polymorphism (denoted rs); *TOMM40*: Translocase of outer mitochondrial membrane 40; WM: White matter.

## Glossary

Allele: One of two versions of a gene, an allele is a DNA coding that occupies a position on a given chromosome.
Amyloid cascade hypothesis: Proposes that the primary pathogenic event in AD is alterations in Amyloid Precursor Protein (APP) leading to the aggregation of the amyloid beta (A*β*) peptide.
Diffusion tensor imaging (DTI): Imaging acquisition that generates three-dimensional representation of the degree and direction of water diffusion (Brownian motion) in each voxel (tensor). Images are derived from computing 3 eigenvalues (*λ*_1_, *λ*_2 _, *λ*_3_) within each tensor.
Fractional anisotropy (FA): DTI measure derived from the ratio of eigenvalues in each voxel, providing information about the directionality of diffusion along a scale of 0 (isotropic diffusion) to 1 (anisotropic diffusion) where 0 represents completely random diffusion indicative of damaged along white matter fibers.
Freesurfer: Software allowing assessment of volumetric and cortical thickness measures of the brain.
Genomewide association study (GWAS): Study by which the whole genome within a population is assessed in an attempt to identify common genetic associations with diseases such as AD.
Linkage disequilibrium (LD): Defines a genetic region that has had minimal recombination through ancestral history, making genes within such regions linked and dependent of each other.
Mean Diffusivity (MD): A DTI measure derived from the mean of the three eigenvalues that reflects magnitude of water diffusion within a voxel, without providing directionality. Increased MD is an indicator of tissue degeneration.
Missing heritability: The notion that a large proportion of the heritability of complex diseases such as AD remains unknown. Current GWAS have been able to identify genes with small effect sizes, leading us to ask where in the genome the remaining heritability is contained and why we cannot observe them with current techniques.
Mitochondria: Are often referred to as the powerhouse of the cells, as they produce adenosine triphosphate (ATP), the main energy source of the cell. Mitochondria have an outer and inner membrane, with the outer being permeable via active import channels allowing the passage of proteins that are essential for ATP. Mitochondria are mainly independent organelles, as they contain their own DNA. While they are the major energy provider to the cells, they are also critical for cell survival, cell division, and neuronal death.
Mitochondrial cascade hypothesis: Proposes that mitochondria are the primary source of pathology in AD, driving A*β* plaque and neurofibrillary tangle formation.
Radial diffusion: Radial diffusivity (*λ*_2_ + *λ*_3_) represents perpendicular diffusion across fiber pathways. Reduced radial diffusion has been associated with myelin damage.
Reactive oxygen species (ROS): A term that describes a variety of byproducts that are formed during the metabolism of oxygen, otherwise known as free radicals. In mitochondria they are formed as a result of respiration, and a disruption in this balance by increased ROS within mitochondria negatively influences cell survival.
Region of interest (ROI): Imaging procedure that involves manual outlining of an a priori brain region for volumetric analysis.
Retrogenesis: The hypothesis that brain areas that were early to develop are also the first to show AD-type pathology.
Single nucleotide polymorphism (SNP): Stands for a difference in DNA sequence, occurring at a single nucleotide (A, T, C, or G) on a paired chromosome in an individual and denoted by RS (related sequence).
Translocase of outer mitochondrial membrane (TOM): A major import channel on the outer mitochondrial membrane that allows for the influx of proteins to the intermembrane space of the organelle. Tom40 is the main component of this channel serving as the only channel by which proteins enter mitochondria.
Voxel Based Morphometry (VBM): Automatic procedure that allows whole-brain voxelwise analysis of tissue density and volume.
Wallerian Degeneration: Is a model for axonal degeneration. Initial neuronal damage is hypothesized to result in distal axonal degeneration.
